# Unveiling Ag-Modulated Cu Active Sites for Enhanced Multicarbon Product Formation in CO_2_ Electroreduction

**DOI:** 10.1021/acs.jpclett.5c01788

**Published:** 2025-08-26

**Authors:** Felicia Di Costola, Nicolò B. D. Monti, Ilargi Napal, Elena Magnano, Candido F. Pirri, Giancarlo Cicero, Marco Fontana, Francesca Risplendi, Silvia Nappini, Juqin Zeng

**Affiliations:** † Department of Applied Science and Technology (DISAT), 19032 Politecnico di Torino, Corso Duca degli Abruzzi 24, Turin 10129, Italy; ‡ 518735 CNR - Istituto Officina dei Materiali (IOM), Trieste, Basovizza 34149, Italy; § 121451 Istituto Italiano di Tecnologia - IIT, Centre for Sustainable Future Technologies (CSFT), Via Livorno 60, Turin 10144, Italy; ∥ Universitá degli Studi di Trieste, Physics Department, P.le Europa 1, 34127 Trieste, Italy

## Abstract

The development of innovative electrocatalysts for CO_2_ reduction reaction (CO_2_RR) is essential for producing high-value chemicals and fuels. Here, we report a simple surfactant- and solvent-free strategy to fabricate Cu–Ag bimetallic gas diffusion electrodes (GDEs) via sputtering of Cu onto a carbon substrate, followed by galvanic replacement with Ag. This method yields highly pure and tunable electrodes with minimal processing steps. The resulting CuAg GDEs exhibit a marked enhancement in CO_2_RR performance compared to monometallic Cu, particularly in promoting C_2_ (mainly ethanol and ethylene) product formation. This improvement is most pronounced when the galvanic replacement is carried out at 75 °C, yielding an optimal Ag/Cu ratio that maximizes electrochemical performance. Under these optimized conditions, Faradaic efficiencies (FE) for C_2_ products reach 73% and 69% at high current densities of 400 and 600 mA cm^–2^, respectively. Notably, the introduction of Ag markedly improves operational stability, with the system maintaining a FE of 49% for C_2_ products after 3 h of continuous electrolysis. In situ X-ray absorption spectroscopy (XAS) reveals that Ag plays a key role in stabilizing of Cu^+^ species under reaction conditions, which correlates with the C–C coupling and long-term selectivity. These findings provide valuable insights for rational design of advanced Cu-based catalysts for high-performance CO_2_ conversion.

## Graphical abstract

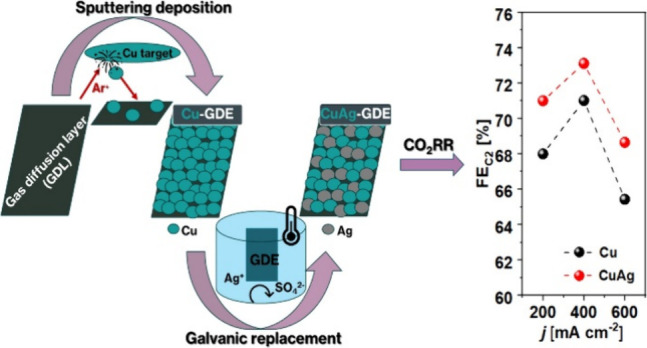

The transformation of CO_2_ into value-added products such as chemicals and fuels using renewable electricity through electrochemical CO_2_ reduction (CO_2_RR), is an increasingly promising strategy to mitigate global climate change and transition toward sustainable energy systems.^1,2^ Despite its potential, CO_2_RR remains limited by significant thermodynamic and kinetic barriers, arising from the high stability of the CO_2_ molecule (C=O bond energy ≈ 750 kJ/mol), and the complexity of multielectron/proton transfer steps in aqueous electrolytes. These factors often result in low energy efficiencies and poor product selectivity. Hence, the development of highly active, selective, and stable electrocatalysts is critical to enabling efficient CO_2_ conversion.^3–6^ Among the various materials investigated, copper (Cu) remains the only monometal capable of catalyzing the formation of C_2_ products—such as ethylene and ethanol—which are both energy-dense and industrially relevant. However, the products formed on Cu span a wide range, reflecting poor selectivity for a specific hydrocarbon or alcohol product.^7–23^ To overcome these limitations, numerous structural and compositional modifications have been explored, with bimetallic systems emerging as a particularly effective strategy.^24–27^ The CuAg bimetallic catalysts stand out due to their proven CO_2_RR performance,^28–43^ stemming from (i) the tandem effect, where Ag promotes CO formation and Cu promotes subsequent C–C coupling,^32,38,43^ and (ii) the electronic effect, where Ag alters the electronic structure of Cu, thereby modulating the binding energy of key intermediates.^29,30,34,35,37^ Despite these advantages, the fabrication of CuAg catalysts often relies on complex synthetic routes, which may hinder scalability and reproducibility. In addition, the influence of Ag on the long-term stability of these bimetallic systems remains poorly understood, warranting further investigation.

In this work, we present a simple and scalable tow-step strategy for the fabrication of CuAg bimetallic GDEs, as illustrated in Figure S1: (1) sputtering deposition of Cu onto a gas diffusion layer (GDL) substrate,^44^ followed by (2) galvanic replacement of surface Cu atoms with Ag using an aqueous Ag_2_SO_4_ solution.^45–47^ This solvent- and surfactant-free process yields free-standing GDEs that are immediately compatible with high-performance CO_2_ electrolyzer configurations, including flow cells and membrane electrode assembly (MEA) cells. The first sputtering step results in a mass loading of about 170 μg_Cu_/cm^2^. Subsequently, controlled galvanic replacement (eq S1) was performed by immersing Cu-coated GDLs in Ag^+^ solution for 5 min under varying temperatures of 25 °C, 50 °C, 75 and 90 °C, yielding a series of samples labeled CuAg-x, where x represents the temperature in Celsius degrees.

Field emission scanning electron microscopy (FESEM) micrographs show the morphology of the bare Cu and CuAg electrodes (Figure 1 and Figures S2–S8). After a sputtering step, the GDL substrate is uniformly covered by the Cu species (Figure 1a). The surface conformation of the Cu electrode (Figure 1b) appears to exhibit high microroughness and porosity, suggesting a high specific surface area. As more clearly observed from Figure S2, Cu nanoclusters assembled into NPs, some of which further coalesce to form submicrometric particles. The CuAg electrodes display a morphology distinct from that of bare Cu. The morphological differences become increasingly evident at elevated replacement temperatures. On the CuAg-25 sample, nanosheets are nonuniformly distributed over the NPs, which still preserve the characteristic morphology of Cu NPs (Figure 1c and Figure S3). Energy-dispersive X-ray spectroscopy (EDX) analysis reveals that the nanosheets are rich in Ag, while the NPs are mainly composed of Cu (Figure S4). As the replacement temperature increases to 50 °C, the Ag-rich nanosheets evolve into nanoflakes, as observed in the CuAg-50 sample (Figure 1d and Figure S5). The flakes remain nonuniformly distributed across NP layer and exhibit random orientation. Moreover, the morphology of the NPs differs from that of the Cu NPs, characterized by smoother surfaces, more defined boundaries, and reduced particle size. These morphological changes are attributed to the interaction between Cu and Ag species, as the bare Cu electrode maintains a stable morphology throughout the temperature range investigated in this work. A further increase in replacement temperature leads to more pronounced changes in the sample morphology. The surface of the CuAg-75 sample is primarily composed of nanoflakes, with some NPs growing on top of them (Figure 1e and Figure S6). Both the nanoflakes and NPs are rich in Ag species, confirming that these NPs form during the displacement reaction (Figure S7). The CuAg-90 sample shows a similar morphology to CuAg-75, with more evident NP growth on the surface (Figure 1f and Figure S8). In addition to morphology, the effect of temperature is also reflected in the Ag/Cu ratio obtained from EDX analysis, which increases progressively from 0.14 to 0.26, 0.70, and 0.80 for CuAg-25, CuAg-50, CuAg-75 and CuAg-90, respectively (Table S1). A significant increase in this ratio occurs between 50 and 75 °C, aligning with the morphological analysis, which reveals a notable transition at 75 °C. These findings suggest that the replacement kinetics accelerate with raising temperature and with a critical threshold between 50 and 75 °C.

**Figure 1:**
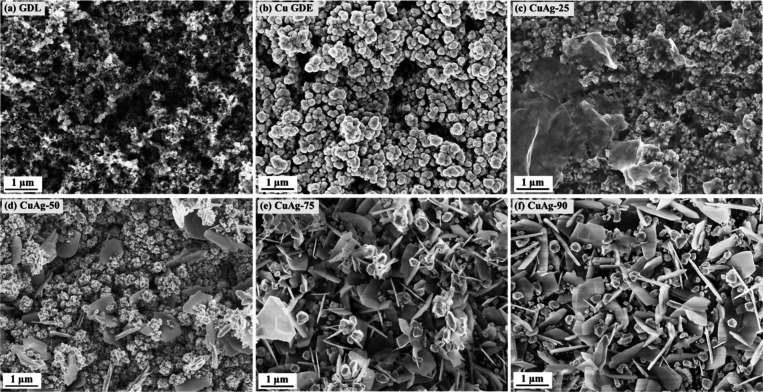
FESEM images of the samples: (a) GDL substrate, (b) Cu gas diffusion electrode, (c) CuAg-25, (d) CuAg-50, (e) CuAg-75 and (f) CuAg-90.

Figure 2 shows X-ray diffraction (XRD) patterns of various samples. It is surprising to observe a close match between the diffraction patterns of Cu GDE and GDL substrate, with no detectable Cu species. These results may be attributed to the extremely small size (<2 nm) of Cu clusters as the primary particles, as revealed by FESEM analysis, or to the low crystallinity. The CuAg-25 shows a similar diffraction pattern to that of the Cu GDE, except for a weak diffraction peak at 38.3°, corresponding to the Ag (111) lattice plane (PDF#00-004-0783). As the replacement temperature increases to 50 °C, the diffraction peaks related to the Ag crystalline phase become more pronounced, reflecting the higher Ag content on the surface. Additionally, peaks corresponding to the Cu_2_O crystalline phase (PDF#00-005-0667) appear, likely due to the formation of larger crystalline domains in the CuAg-50. FESEM analysis further supports this observation, indicating NP growth that may result from Ostwald ripening process.^48^ During the replacement reaction, many Cu clusters may detach from the aggregates formed during the sputtering deposition. Due to the high solubility and increased surface energy, smaller clusters tend to redissolve into the solution, facilitating the growth of larger particles. A further increase in the replacement temperature results in a significant enhancement in the intensity of the diffraction peaks corresponding to the Ag phase, while the peaks associated with Cu_2_O decrease in intensity. This trend indicates a higher Ag content and a reduction in Cu within the electrode, as expected. At higher displacement temperatures, additional diffraction peaks emerge, corresponding to two mixed oxide phases: Ag_2_Cu_2_O_3_ (PDF#97-005-1672) and AgCuO_2_ (PDF#96-150-9290).

**Figure 2:**
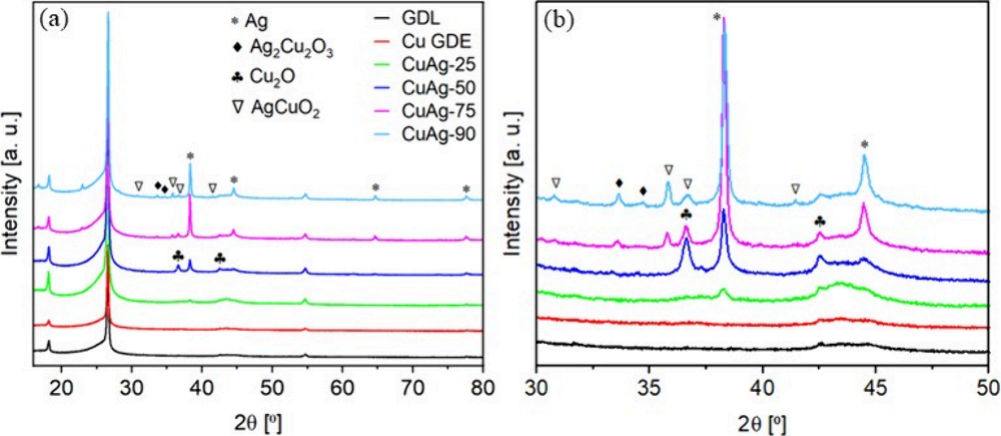
XRD patterns for GDL, Cu electrode and the four bimetallic catalysts synthesized at (a) different temperatures and (b) zoom of the patterns in the 30–50° range of 2θ.

Ex-situ X-ray absorption spectroscopy (XAS) and X-ray photoelectron spectroscopy (XPS) were employed to gain deeper insights into the surface composition of the synthesized materials. Figure 3 presents the XAS spectra at the Cu L-edge and O K-edge for the investigated samples, along with reference spectra of metallic Cu, Cu_2_O, and CuO, all acquired in fluorescence yield (FY) mode. To complement these measurements, the same spectra were also recorded in total electron yield (TEY) mode (Figure S9), which offers significantly higher surface sensitivity, probing depths of only a few nanometers, compared to the tens of micrometers typical of FY mode.

**Figure 3:**
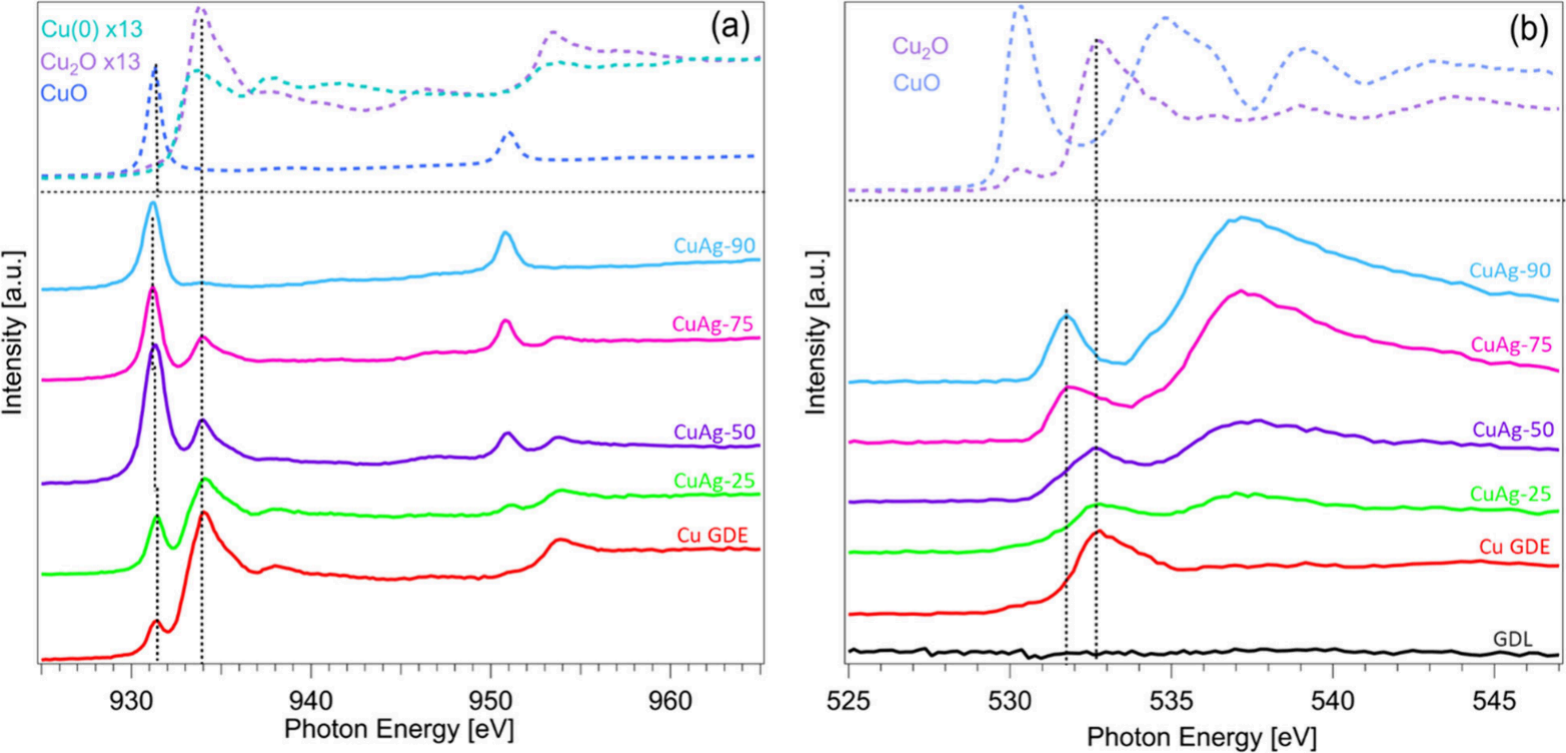
(a) Cu L-edge XAS and (b) O K-edge XAS spectra of Cu, CuAg-25, CuAg-50, CuAg-75, and CuAg-90 samples prepared on GDL, all measured in FY mode. For comparison, the Cu L-edge and O K-edge reference spectra of metallic Cu, Cu_2_O, and CuO are also shown.

The Cu L- and O K-edge spectra indicate that the Cu GDE sample is composed of approximately 2 ± 0.2% CuO, 55 ± 3% Cu_2_O, and 43 ± 2% metallic Cu. Following the introduction of Ag at 25 °C, an increase in the intensity of the spectral feature at 931.3 eV, associated with Cu­(II) species, primarily CuO, is observed. The intensity of this feature grows further with increasing temperature, suggesting progressive increase of Cu­(II) in the CuAg sample prepared at a higher temperature. Notably, a gradual downshift of approximately 0.1 eV in the Cu­(II)-related feature is observed as the temperature increases from 25 to 90 °C. This energy shift is indicative of a modification in the local chemical environment around Cu atoms, likely due to the interaction with Ag atoms. This interpretation is further supported by the O K-edge XAS spectra. While the initial Cu GDE sample shows a dominant Cu_2_O peak at ∼532.8 eV, this feature broadens with Ag incorporation and increasing temperature, indicating changes in the local bonding structure. Additionally, a new spectral component emerges at ∼531.8 eV, which is distinct from the typical CuO feature at ∼530.4 eV and from those associated with Ag_2_O or Ag_3_O_4_, which are also typically observed around ∼530.4 eV.^49–51^ This new feature likely reflects a strong chemical interaction between Ag and Cu species, suggesting the formation of a mixed Ag–Cu oxide phase at temperatures higher than 75 °C. The XPS analysis, presented in Figure S10, confirms the expected elemental composition and is consistent with the results obtained from the XAS measurements. A progressive increase in the Cu­(II) contribution is evident in both the Cu 2p core-level and Cu LMM Auger spectra as the preparation temperature rises. These changes cannot be ascribed solely to the formation of a pure CuO phase, but rather suggest enhanced surface interactions between Cu­(II) species and Ag atoms. This evolution is consistent with the formation of Ag-rich surface layer in intimate contact with the Cu oxide framework followed by the progressive chemical incorporation of the Ag atoms in the bulk, ultimately forming a mixed Ag–Cu oxide phase above 75 °C.^52,53^ This temperature-dependent evolution is also supported by Ag 3d core level and Ag MNN Auger spectra. Specifically, at lower temperatures (≤90 °C), the Ag 3d peaks in the CuAg samples are shifted by approximately 0.1 eV to lower binding energies compared to those of pure Ag or Ag_2_O. Up to 75 °C, no significant changes in peak shape or binding energy position are observed, suggesting the coexistence of metallic Ag and/or Ag_2_O phases^54,55^ in contact with Cu oxide nanostructures.^53^ However, at the highest investigated temperature, a pronounced broadening and energy shift of both Ag 3d and Ag MNN Auger features emerges, suggesting the formation of a new and less conductive Ag-containing phase. These spectral changes are consistent with the development of a mixed Ag–Cu oxide phase, likely arising from enhanced chemical interaction between Ag and Cu species at elevated temperatures.

Although direct signatures of a mixed Ag–Cu oxide phase are not clearly resolved in the Ag spectra alone, complementary evidence from Cu 2p, Cu LMM Auger, Ag 3d, Ag MNN Auger, as well as Cu L-edge and O K-edge XAS measurements, indicate that such a phase begins to form at the surface above 50 °C becoming more structurally integrated and prominent within the bulk at higher temperature. The apparent absence of Ag-related spectral evolution at lower temperatures can be attributed to the higher bulk sensitivity of the Ag 3d XPS signal, which results from the higher kinetic energy of the emitted photoelectrons at the employed photon energy, compared to the Cu 2p signals and XAS data collected in both FY and TEY modes.

The differences observed across various characterization techniques underscore the importance of combining surface- and bulk-sensitive methods to comprehensively understand the structural evolution of the Cu–Ag system.

Concisely, both Cu and CuAg samples exhibit complex compositions. The Cu GDE consists of CuO, Cu_2_O, and metallic Cu, as revealed by XPS and XAS analyses; however, these phases are not detected in XRD pattern, likely due to the ultrasmall size of the NPs observed via FESEM or the poor crystallinity of these phases. All CuAg samples show the presence of metallic Ag, with its proportion increasing at higher replacement temperatures. Above 50 °C, Cu–Ag mixed oxides emerge—initially detected on the surface of the CuAg-50 sample by XPS and subsequently observed in both the surface and bulk of CuAg-75 and CuAg-90 samples, as confirmed by XRD, XPS and XAS analyses.

The CO_2_RR performance of CuAg GDEs was evaluated in a flow cell with 1 M KOH electrolyte (schematically shown in Figure S11) with galvanostatic measurements. For comparison, Cu GDEs were also tested, exhibiting good selectivity toward C_2_H_4_ and ethanol production in the investigated current density range from 200 to 600 mA cm^–2^ (Figure S12a), consistent with previous reports.^10,19–21^ The selectivity toward both ethanol and C_2_H_4_ can be improved by using CuAg electrodes, depending on their composition (Figures S12b-d and Figure 4a). Ethanol production is favored at low Ag/Cu ratios, whereas higher ratios tend to promote C_2_H_4_ formation. On low Ag-loaded CuAg-25 and CuAg-50 GDEs, ethanol selectivity increases by 4–10%, while the C_2_H_4_ selectivity drops significantly. In contrast, CuAg-90—with the highest Ag/Cu ratio—shows reduced ethanol selectivity but maintains good selectivity toward C_2_H_4_ (Figure S13). Among the tested samples, only CuAg-75 exhibits enhanced selectivity for both ethanol and C_2_H_4_ compared to pure Cu GDEs across most of the investigated current densities. Consequently, the overall selectivity for C_2_ products is significantly improved on the CuAg-75 electrode, reaching a maximum of 73% at 400 mA cm^–2^, corresponding to a partial current density of 292 mA cm^–2^, mainly for ethanol and C_2_H_4_ production (Figure 4b). Several studies have shown that increasing the Ag content in Ag/Cu catalysts generally decreases C_2_H_4_ selectivity, while ethanol production initially increases at low Ag/Cu ratios and then declines as Ag concent continues to rise.^28,32,40,41^ These findings suggest the existence of an optimal Ag/Cu ratio that promotes ethanol formation, consistent with our observations. In contrast, other reports have indicated that the presence of Ag enhances only C_2_H_4_ production on Cu-based catalysts.^29,42^ This inconsistency across the literature highlights the significant challenge in identifying the key factors governing the Cu–Ag synergy, including chemical composition, structural rearrangements, and oxidation state.

**Figure 4:**
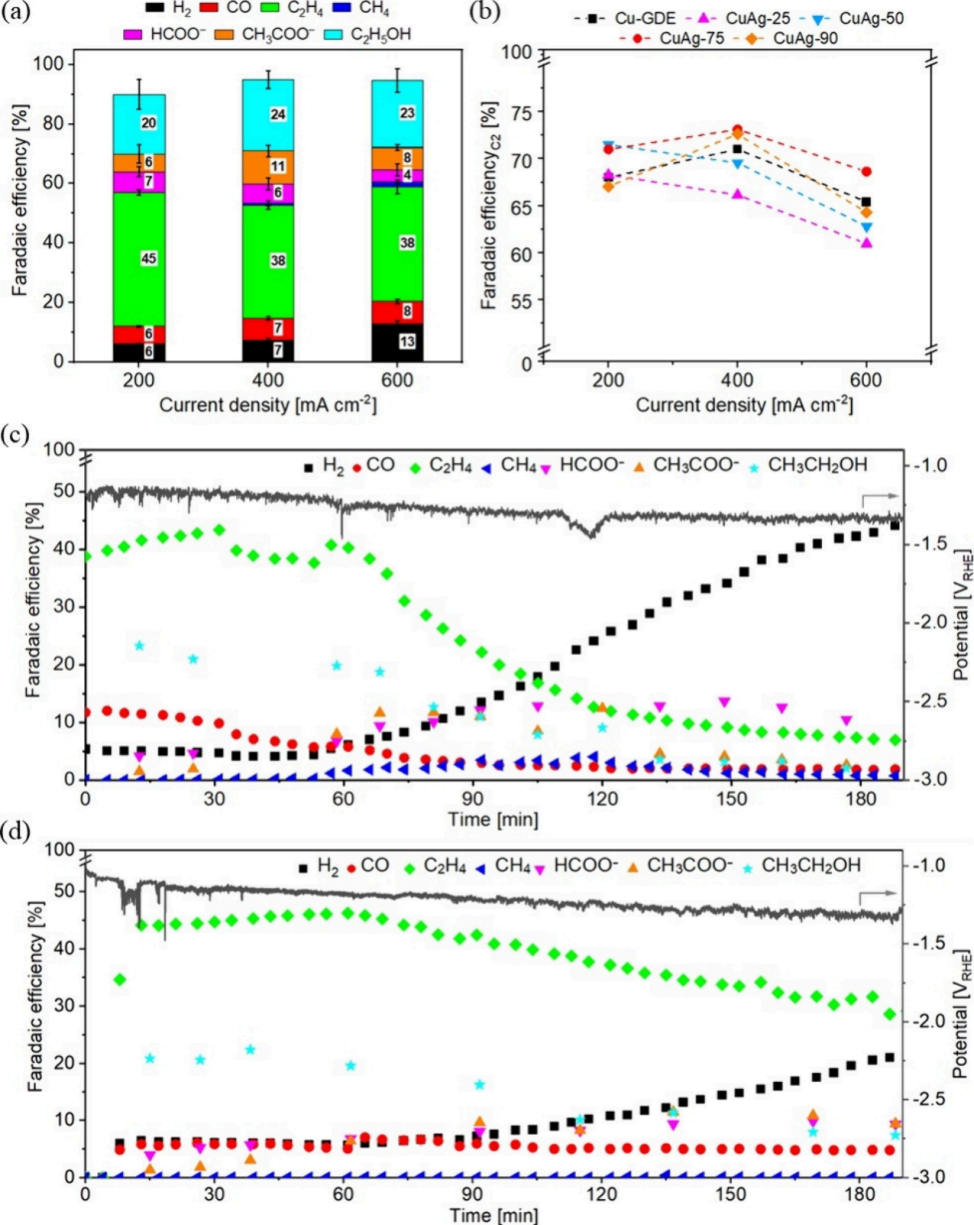
(a) FE values for 1h-test in a flow cell setup at various current densities for CuAg-75. (The values presented in the histograms represent the average obtained from three repetitions of the same test, while the error bars correspond to the standard deviation.) (b) Comparison of the FE for C_2_ production across all the investigated catalysts and resulting FE and corresponding potentials over long-term test for (c) Cu-GDE and (d) CuAg-75 GDE in the flow cell setup. The reported potentials are not corrected for *iR* drop.

Long-term tests were further conducted at a current density of 200 mA cm^–2^ in the flow cell for 3 h to compare the stability of the Cu GDE (Figure 4c) and the best-performing CuAg-75 electrode (Figure 4d). After an initial hour of stable gas production on both electrodes, a notable difference emerges in H_2_ evolution, which increases more rapidly for Cu GDE than for CuAg-75. The CuAg-75 catalyst demonstrates greater stability, preserving C_2_H_4_ selectivity for a longer duration and maintaining more stable CO production over time. These results suggest that the addition of Ag not only enhances C_2_ product formation but also plays a crucial role in improving catalyst stability during the CO_2_RR process.

To assess also the physicochemical stability of the material obtained at 75 °C, the morphological characterization by FESEM was conducted at different stages of the electrochemical measurement (after 1 and 3 h of the test). As shown in Figure S14, it is evident that the electrode undergoes a noticeable morphological chang after 1 h of operation (Figure S14b and S14e) compared with the pristine one (Figure S14a and S14d). The surface appears more nanostructured, partially covering the typical flake-like features associated with the galvanic displacement of Ag observed in the fresh catalyst layer. However, no further significant morphological changes are observed during the prolonged test (Figure S14c and S14f). This outcome suggests that the material is likely to restructure during the initial stage of catalysis, as commonly reported for Cu-based systems under CO_2_RR conditions.^44,56–59^ In contrast, the changes in product selectivity emerge after 1 h of testing. Hence, it is reasonable to infer that the observed morphological changes are not essentially correlated with the shift in electrochemical performance. Not only does the morphology remain stable after 1 h, but the Ag/Cu ratio also stays constant throughout the long-term test, as confirmed by EDX analysis. Therefore, the performance degradation of CuAg-75 during the test is likely attributed to flooding issues, commonly observed in flow cell setups,^60,61^ rather than to material failure.

Since the CO_2_ catalysts are widely reported to undergo significant restructuring under reductive conditions,^62,63^ the properties of the as-prepared samples cannot be directly correlated with their CO_2_RR performance. To investigate the real synergy between Cu and Ag, in situ soft X-ray absorption spectroscopy (s-XAS) analysis was performed on both CuAg and Cu electrodes under CO_2_RR conditions using a batch electrochemical cell (Figure S15). The Cu L-edge spectra were acquired as a function of the applied potential, with two consecutive scans collected 15 min apart, providing insights into the unoccupied electronic states of Cu under working conditions relevant to the CO_2_RR. A quantitative analysis of the operando s-XAS spectra was conducted by deconvoluting the experimental spectra (2nd scan) using reference Cu L-edge spectra, corrected for the corresponding absorption cross sections (see Figure 3a), to monitor changes in the Cu redox state as a function of potential. Importantly, the transition of the Cu oxidation state at each applied potential does not occur instantaneously but requires at least 15 min to stabilize, highlighting the time-dependent nature of the redox processes under operando conditions.

The operando s-XAS spectra, shown in Figure 5, reveal that at open circuit potential (OCP, −0.26 V vs Ag/AgCl), the Cu electrode predominantly exists in the Cu­(I) state (70 ± 3%), with minor contributions from Cu(0) (28 ± 1%) and Cu­(II) (2 ± 0.1%). In contrast, the bimetallic CuAg-75 catalyst displays a mixture of 80 ± 4% Cu­(I) and 20 ± 1% Cu­(II) under the same conditions. Upon applying progressively more negative potentials, a gradual reduction of the Cu oxidation state is observed in both samples, as illustrated in Figure 5c and 5d for Cu and CuAg-75, respectively. Notably, the bimetallic CuAg-75 catalyst retains a higher proportion of Cu­(I) (95% ± 4.5%) even at – 0.75 V vs Ag/AgCl, a potential at which the pure Cu electrode is almost fully reduced to metallic Cu(0). This clearly indicates that the presence of Ag stabilizes higher oxidation states of Cu under CO_2_RR-relevant conditions. Additionally, after the operando s-XAS experiments, when both samples were left to return to OCP, the Cu electrode remained predominantly in the metallic state, with only a minimal reformation of Cu­(I), whereas the CuAg-75 catalyst reverted entirely to the Cu­(I) oxidation state (Figure S16, Supporting Information). This further confirms that Ag promotes the stabilization of Cu­(I) species. No significant changes were detected in the O K-edge spectra for the two investigated samples (Figure S17, Supporting Information), likely due to strong absorption from atmospheric contaminants on the Si_3_N_4_ window, which hindered the oxygen-related features in the samples.

**Figure 5:**
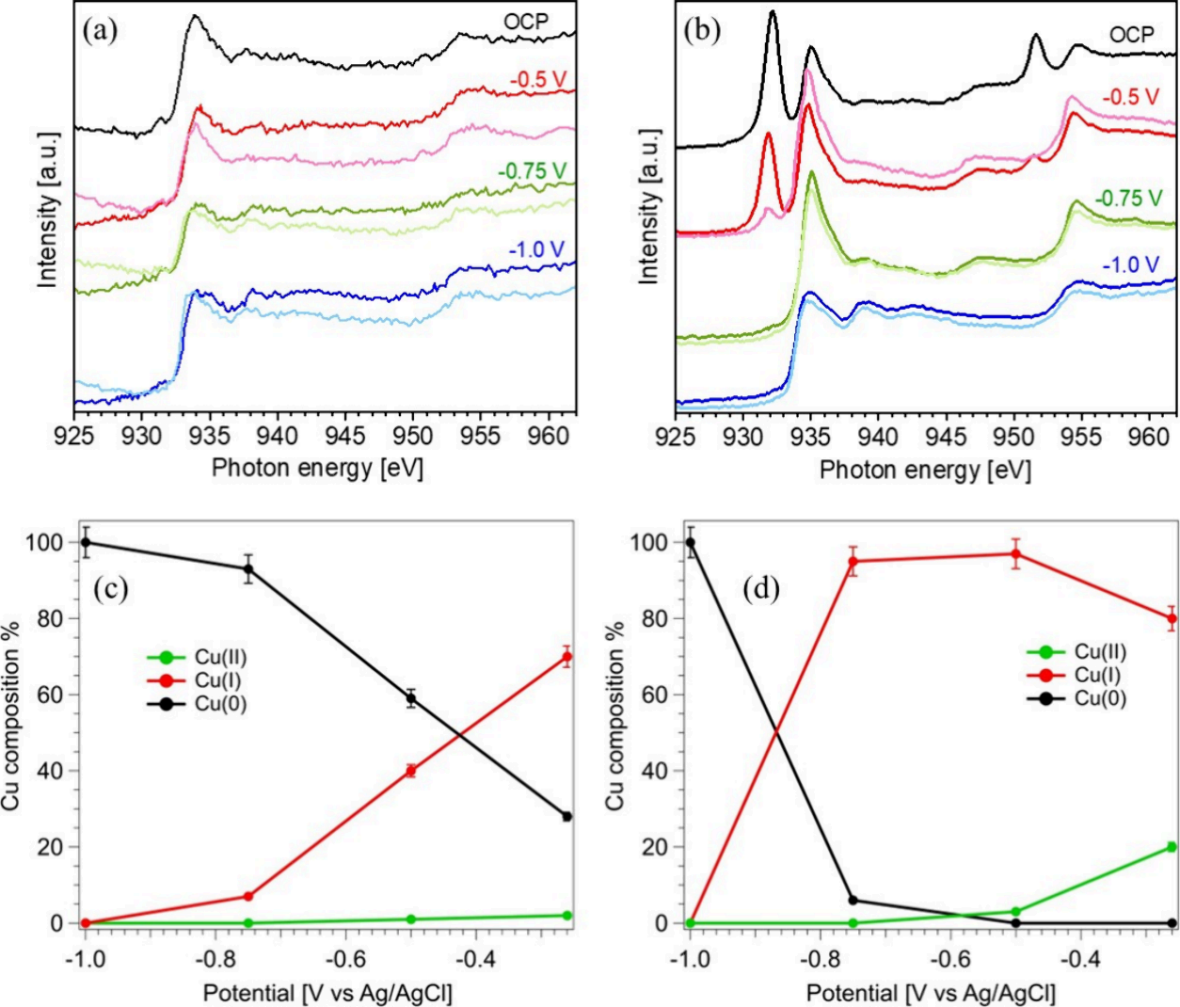
Cu L-edge XAS spectra recorded in FY mode from OCP to −1 V vs Ag/AgCl for (a) Cu and (b) CuAg-75 prepared on Au-coated Si_3_N_4_ window. Evolution of the Cu oxidation state as a function of the applied potential for (c) Cu and (d) CuAg-75.

By correlating operando measurements with the electrochemical characterization of Cu and CuAg-75 electrodes, the role of Ag in enhancing the catalytic performance of the CuAg system for CO_2_RR can be elucidated. Due to the more electronegative nature, Ag can withdraw electron density from neighboring Cu atoms, leading to the persistence of Cu­(I) species, as observed by operando XAS analysis. Such stabilization is crucial, as Cu­(I) has been widely correlated with enhanced selectivity toward C_2_ products in CO_2_RR. Cu metal plays a key role in CO_2_ activation and facilitates subsequent electron transfer steps, while oxidized Cu species enhance *CO adsorption, promoting C–C coupling.^64–66^ Density functional theory (DFT) calculations^67^ and recent experimental studies using infrared spectroscopy and in situ XAS^68^ further support this mechanism, suggesting that the asymmetry in CO adsorption energies between metallic and oxidized Cu sites facilitates CO dimerization that is a crucial step for C_2_ product formation. Based on our experimental results and consistent literature evidence, we conclude that the persistence of Cu cations under in the presence of Ag is closely linked to the formation of stable C_2_H_4_ products.

In summary, this study aimed to develop a facile synthesis method for producing CuAg bimetallic catalysts. The displacement temperature between copper and silver was found to play a key role in determining the structure and composition of the resulting materials, with higher temperature leading to an increased Ag/Cu ratio. This compositional change significantly influences CO_2_RR performance: lower ratios favor ethanol production, while higher ratios enhance C_2_H_4_ production. Among the samples, the CuAg sample prepared at 75 °C exhibited optimal selectivity toward both ethanol and C_2_H_4_, achieving the best overall C_2_ product selectivity. Compared to the bare Cu electrode, this CuAg catalyst also demonstrated significantly improved stability in C_2_ production. The enhanced CO_2_RR performance is attributed to the more persistence of Cu cationic species under negative potentials in the presence of Ag, as revealed by in situ XAS analysis. These findings highlight a clear correlation between Ag-induced stabilization of Cu cations (Cu^+^), and sustained C_2_ product formation on CuAg electrode. Future work will focus on elucidating the reaction intermediates involed in CO_2_RR on Cu and CuAg electrodes over time, using *operando* and *in situ* techniques. This will provide further insight into the CO_2_RR mechanism responsible for enhanced C_2_ product formation in the presence of Ag.

Beyond demonstrating improved CO_2_RR performance at high current densities, the present study highlights synthesis approaches—galvanic replacement and magnetron sputtering—that are not only inherently scalable but also solvent-free, making them attractive for sustainable and industrially viable catalyst fabrication. Both methods are compatible with roll-to-roll manufacturing of gas diffusion electrodes, supporting their potential for large-scale application. Nevertheless, further efforts are needed to evaluate long-term operational durability beyond a few hours and to assess the performance of CuAg GDEs within complete electrolyzer systems under industrially relevant conditions. Addressing these challenges will be essential for the practical implementation of this catalyst platform.

## Supplementary Material



## Data Availability

The data sets generated during and/or analyzed during the current study are available from the corresponding authors upon reasonable request.

## Notes

Schematic of the Cu–Ag electrode preparation, FESEM images of electrodes at different magnifications, EDX analysis on selected zones of the samples, Cu L-edge XAS and O K-edge XAS spectra of samples prepared on GDL measured in TEY mode, XPS spectra, schematic of the electrochemical flow cell, resulting FE in flow cell, scheme of the EC-cell and measurement setup for in situ s-XAS experiments, Cu L-edge spectra recorded in FY mode after the operando XAS experiment, O K-edge spectra recorded in FY mode during operando XAS experiment, Ag/Cu ratios by EDX (PDF)
