# Supplementary material for: Unveiling Ag-Modulated Cu Active Sites for Enhanced Multicarbon Product Formation in CO2 Electroreduction
Source: J Phys Chem Lett. 2025 Aug 26;16(35):9088–96. doi: 10.1021/acs.jpclett.5c01788 (PMC12415878; doi:10.1021/acs.jpclett.5c01788)
Supplement: Supplementary file 1 [file jz5c01788_si_001.pdf]

Supporting information

Unveiling Ag-Modulated Cu Active Sites for Enhanced Multicarbon Product Formation in CO_2 Electroreduction

Felicia Di Costola[a,c], Nicolò B. D. Monti[a,c], Ilargi Napal[b,d], Elena Magnano[b], Candido F. Pirri[a,c], Giancarlo Cicero[a], Marco Fontana[a,c], Francesca Risplendi[a], Silvia Nappini[b], Juqin Zeng[a,c*]*

[a]Department of Applied Science and Technology (DISAT), Politecnico di Torino, Corso Duca degli Abruzzi 24, Turin,10129, Italy

[b]CNR - Istituto Officina dei Materiali (IOM), Trieste, Basovizza 34149, Italy.

[c]Istituto Italiano di Tecnologia - IIT, Centre for Sustainable Future Technologies (CSFT), Via Livorno 60, Turin,10144, Italy

[d] Universitá degli Studi di Trieste, Physics Department, P.le Europa 1, 34127, Trieste, Italy

[*]Corresponding authors: felicia.dicostola@polito.it; juqin.zeng@polito.it

1.1 Synthesis of Cu-Ag gas diffusion electrodes

Typically, the preparation of Cu-Ag GDEs involved two key steps, as illustrated in Figure S1 and detailed below.

-Step 1: Sputter deposition of Cu on carbon paper. Commercial carbon paper (GDL; SIGRACET 28BC, Ion power GmbH) was coated with a high surface area copper layer using a magnetron sputtering device (Quorum Technologies Ltd., Q150T S). The sputtering process was conducted with a Cu target (99.99%, Φ57 mm x 0.5 mm, Nanovision S.r.l.) at an applied current of 50 mA for 400 seconds. The mass loading of Cu on the GDL was calculated through normalizing the weight difference before and after deposition, by the geometric area of the carbon paper.

-Step 2: Galvanic replacement of Cu with Ag. The Cu-coated GDL was immediately immersed in an aqueous 0.5M Ag_2SO_4 solution (≥99.99%, Sigma-Aldrich). The sample transfer was fast to prevent Cu oxidation due to the air exposure. The galvanic displacement reaction between Cu and Ag^+ ions resulted in the formation of the Ag-Cu bimetallic catalyst. In this step, different solution temperatures were studied. The theoretical principle which rules the galvanic replacement reaction is based on the scale of nobility between dissimilar metals. Ag is more noble than Cu. When Cu is immersed in an electrolyte containing Ag^+ ions, the latter reduce while the former dissolves, as described in the following equation (Eq. S1).

$$Cu + 2Ag^+ \rightarrow Cu^{2+} + 2Ag \qquad \text{(Eq. S1)}$$

After the second step, the obtained Cu-Ag electrodes were washed with deionized water, dried with nitrogen flow, and stored in a glove box (Labstar, Mbraun) to prevent atmospheric contamination.

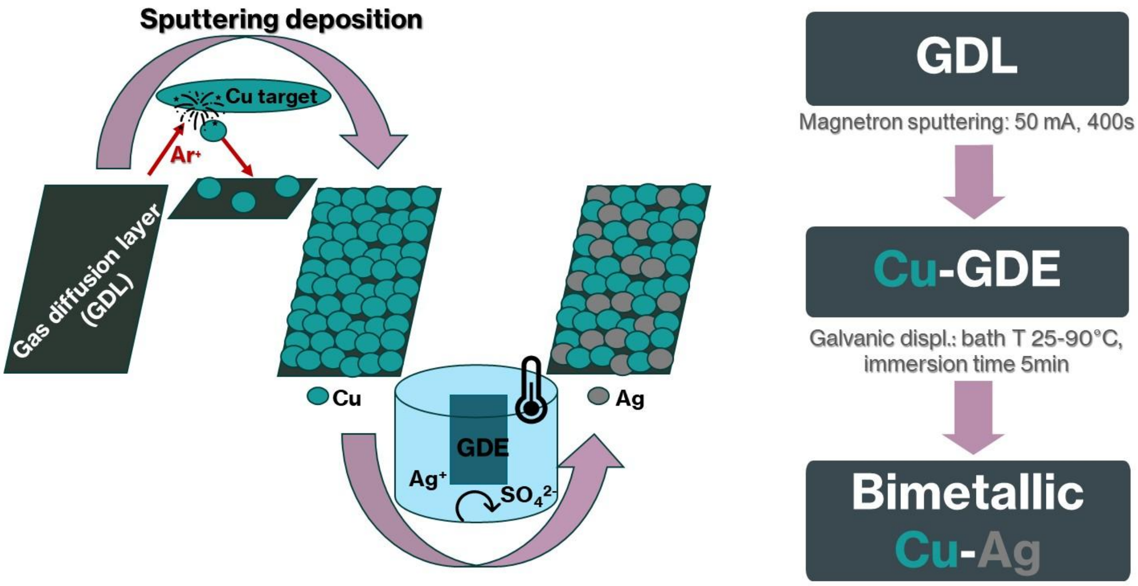

Figure S1. Schematic of the Cu-Ag electrode preparation.

1.2 Ex-situ physicochemical characterization

To understand the physicochemical properties of the prepared materials, a wide range of characterization techniques were employed. The morphological analysis was performed on a Field Emission Scanning Electron Microscope (FESEM, Supra 40, Zeiss, Oberkochen, Germany), equipped with Energy Dispersive Spectroscopy (Oxford EDS microanalysis, Liquid-N_2 cooled Si-Li detector) for qualitative and semi-quantitative chemical composition analysis. The crystalline phases of the samples were analyzed by X-Ray Diffraction (XRD, PANanalytical X'Pert Pro-diffractometer, Cu-Kα radiation, 40 kV, 40 mA, X'celerator detector). X-Ray Photoelectron Spectroscopy (XPS) and XAS characterization of *ex-situ* Cu-Ag GDE samples was performed at the BACH beamline of IOM-CNR at the synchrotron radiation facility Elettra (Trieste, Italy) to understand the overall chemical composition and provide information on the chemical properties of the material surfaces. XAS at the Cu L- and O K-edges were measured in both total electron yield (TEY) mode by detecting the drain current through the sample using a Keithley 428 current amplifier, and in fluorescence yield (FY) mode using a multichannel plate (MCP) detector (Hamamatsu, F4655-13) with an energy resolution better than 0.25eV. These measurements provide insights into the oxidation state of pristine Cu-Ag GDL samples at the surface (TEY) and in the bulk (FY).

To further investigate the surface properties of Cu-Ag samples, XPS measurements were conducted using a hemispherical electron analyzer (Scienta R3000) positioned at an angle of 60° relative to the X-ray incidence direction. Binding energy (BE) values were referenced against the Au $4f_{7/2}$ (84.0 eV) signal from a polycrystalline Au foil in electrical contact with the samples.

XPS spectra, including survey scans, Cu 2p, Ag 3d, Cu LMM, and Ag LMM Auger signals, were recorded using a photon energy of 1144 eV with a total instrumental resolution of 0.4 eV.

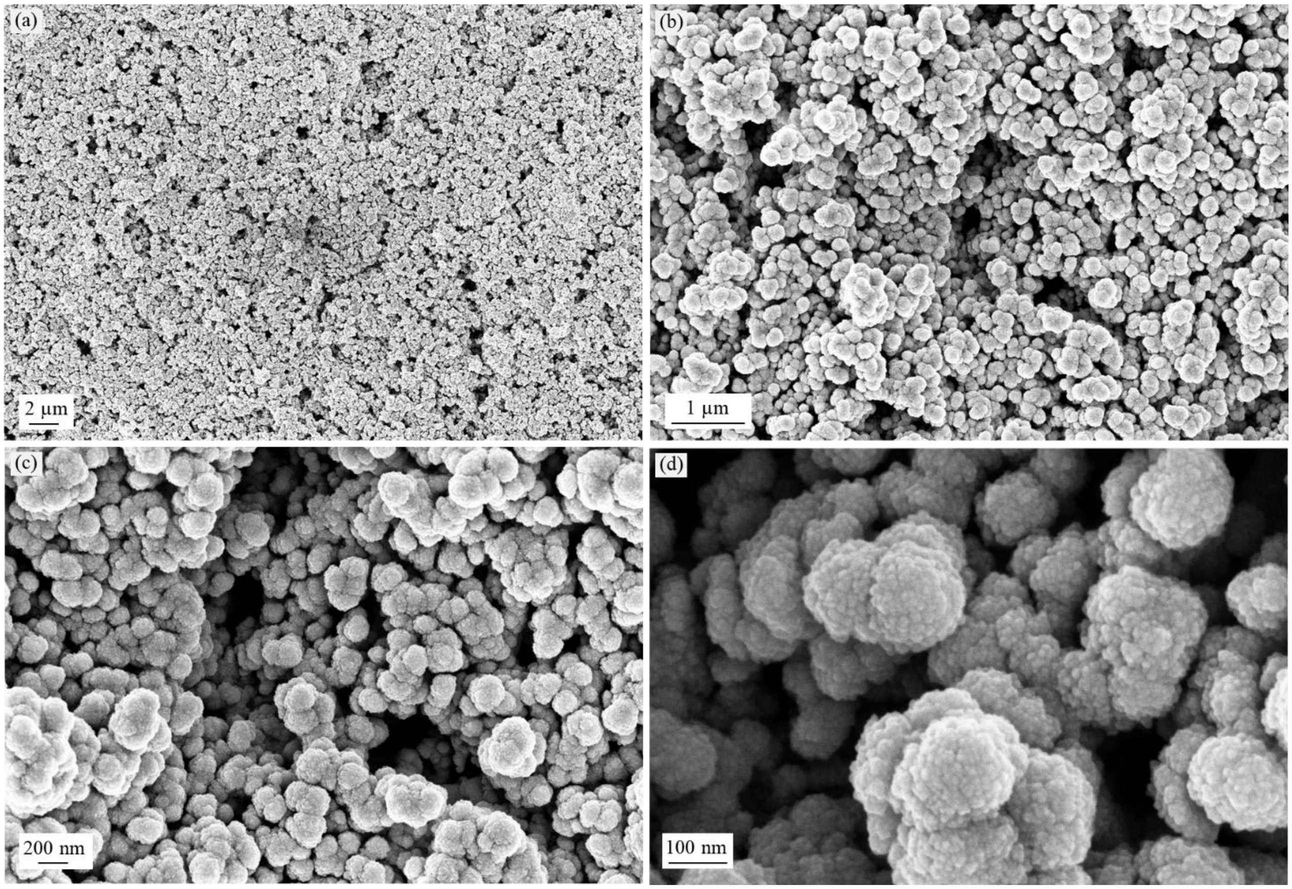

Figure S2. FESEM images of Cu electrode at different magnifications: (a) 5K; (b) 25K; (c) 50K and (d) 200K.

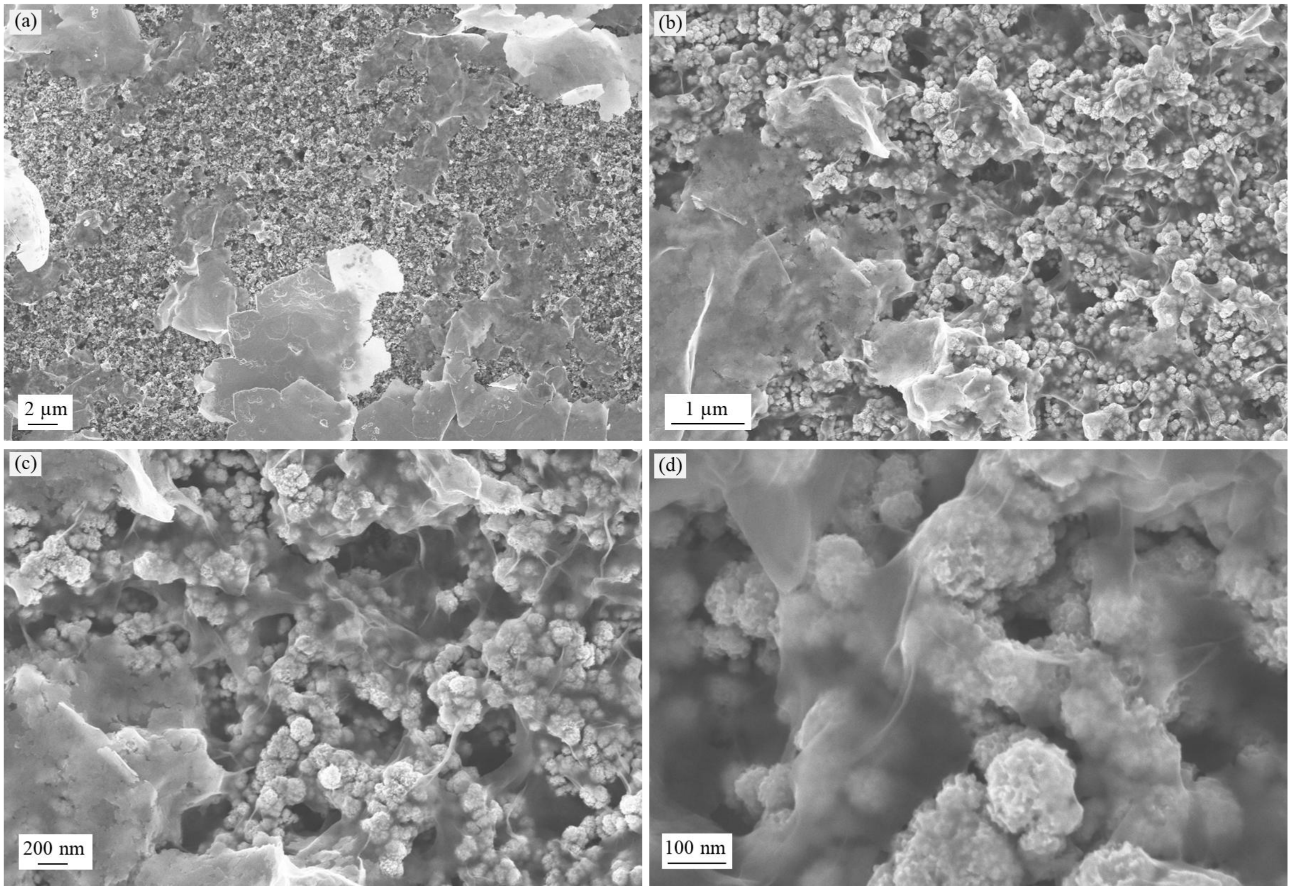

Figure S3. FESEM images of CuAg-25 at different magnifications: (a) 5K; (b) 25K; (c) 50K and (d) 200K.

(a)

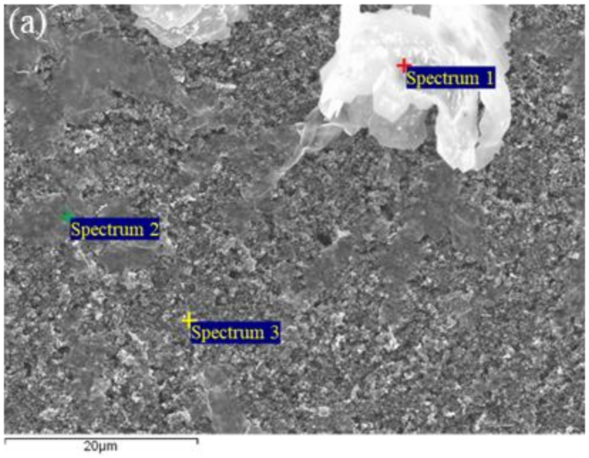

(b)

Zone	+ (red)	+ (green)	+ (yellow)
Element	Atomic %	Atomic %	Atomic %
C	-	72.8	74.3
O	-	5.3	4.2
F	-	11.1	14.1
Cu	7.8	10.0	7.4
Ag	92.2	0.8	-

Figure S4. EDX analysis (15 kV acceleration voltage) on the selected zones of CuAg-25 sample: (a) selected zones and (b) chemical composition of each zone. C and F signals are due to the underlying GDL substrate.

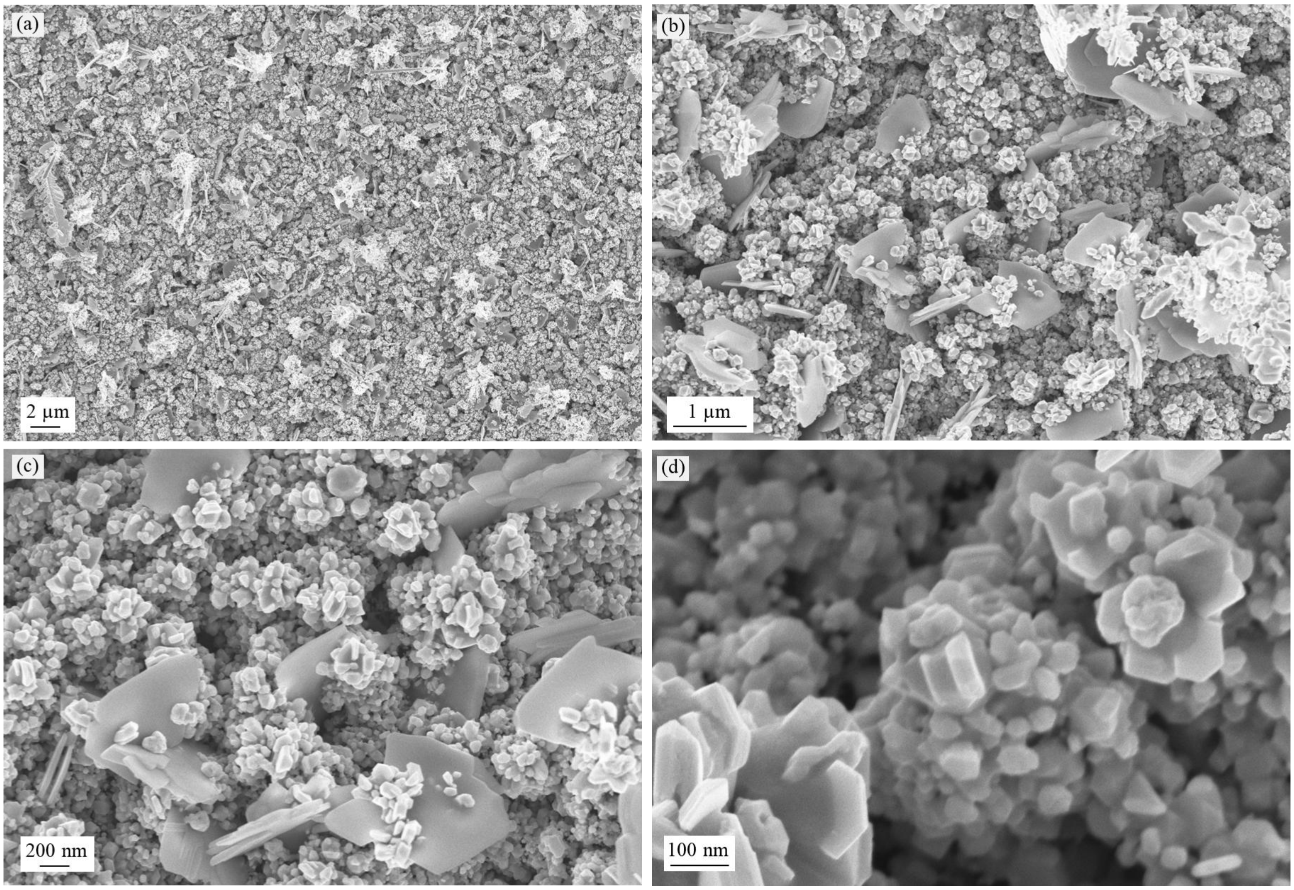

Figure S5. FESEM images of CuAg-50 sample at different magnifications: (a) 5K; (b) 25K; (c) 50K and (d) 200K.

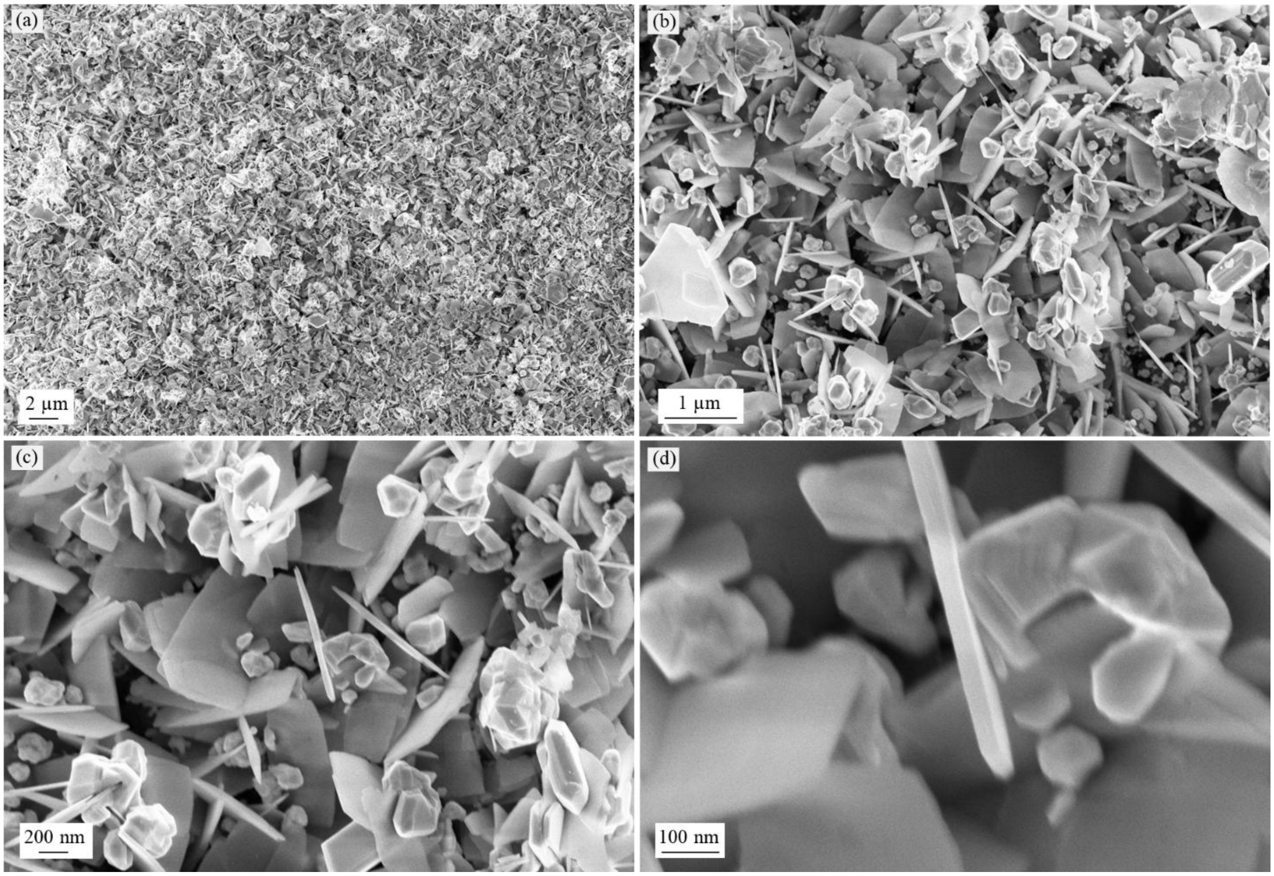

Figure S6. FESEM images of CuAg-75 sample at different magnifications: (a) 5K; (b) 25K; (c) 50K and (d) 200K.

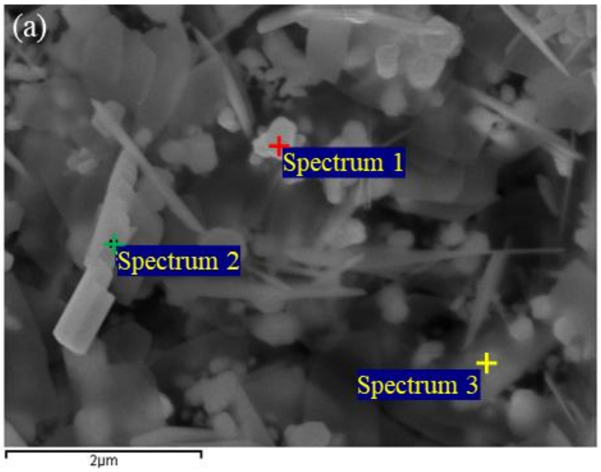

(b)

Zone	+ (red)	+ (green)	+ (yellow)
Element	Atomic %	Atomic %	Atomic %
C	8.6	-	-
O	53.5	56.8	-
F	-	-	-
Cu	14.8	13.1	24.7
S	2.5	1.4	3.6
Ag	20.6	28.7	8.2

Figure S7. EDX analysis (15 kV acceleration voltage) on the selected zones of CuAg-75 sample: (a) selected zones and (b) chemical composition of each zone. The C signal may be ascribed to the underlying GDL substrate or adventitious carbon contamination. S is a residual contamination of the silver sulfate solution involved in the galvanic replacement step.

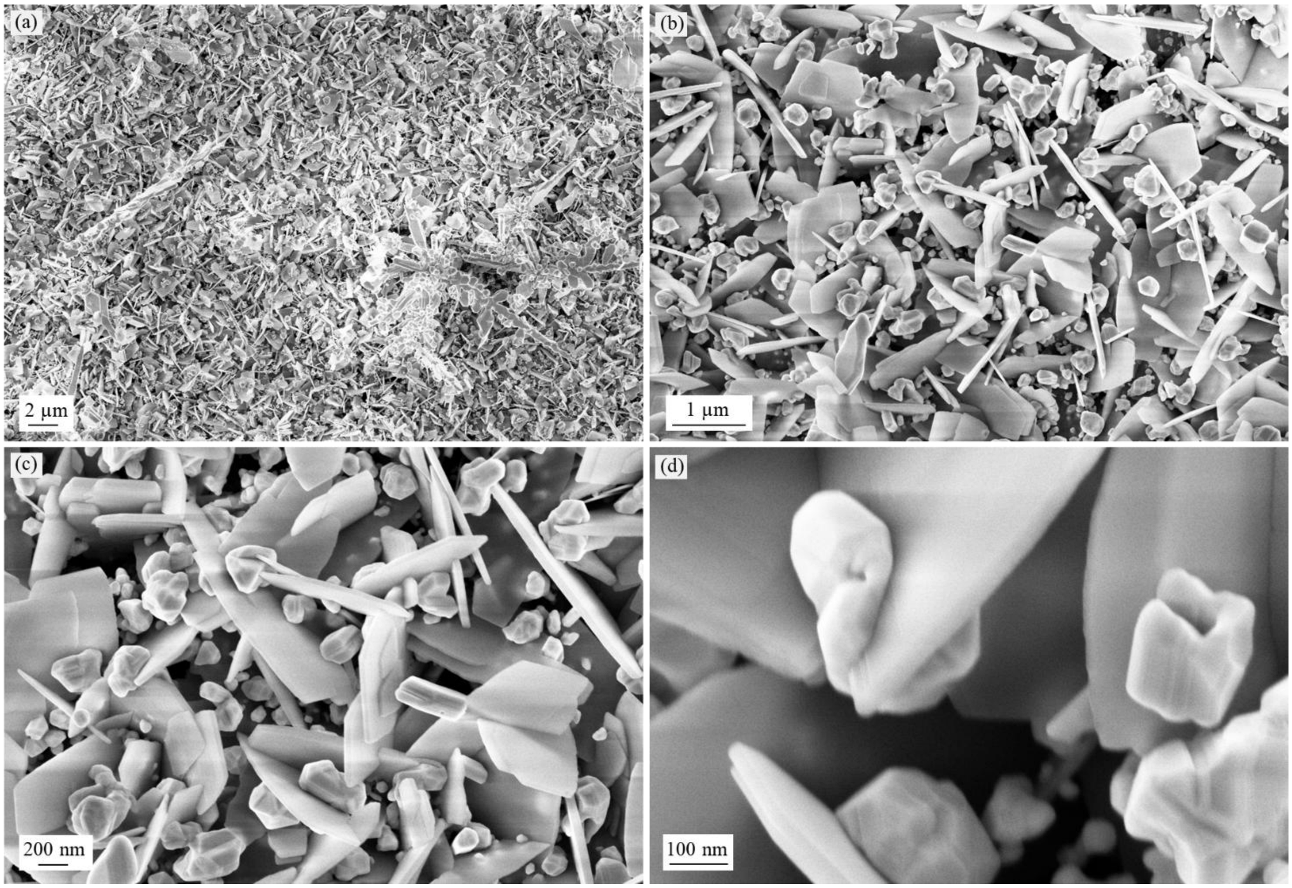

Figure S8. FESEM images of CuAg-90 sample at different magnifications: (a) 5K; (b) 25K; (c) 50K and (d) 200K.

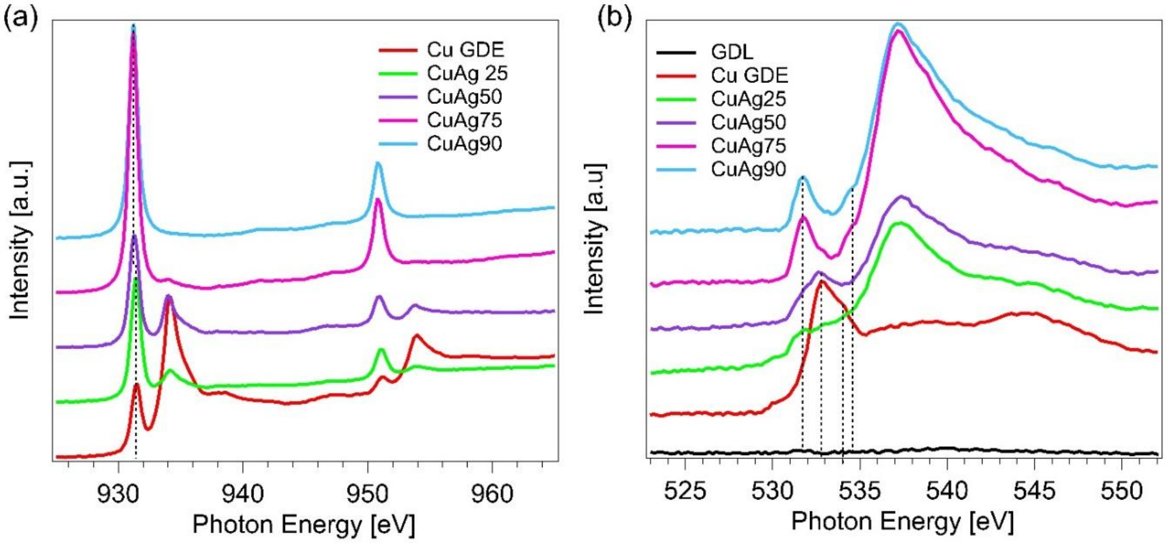

Figure S9. (a) Cu L-edge XAS and (b) O K-edge XAS spectra of Cu, CuAg-25, CuAg-50, CuAg-75, and CuAg-90 samples prepared on GDL, all measured in TEY mode.

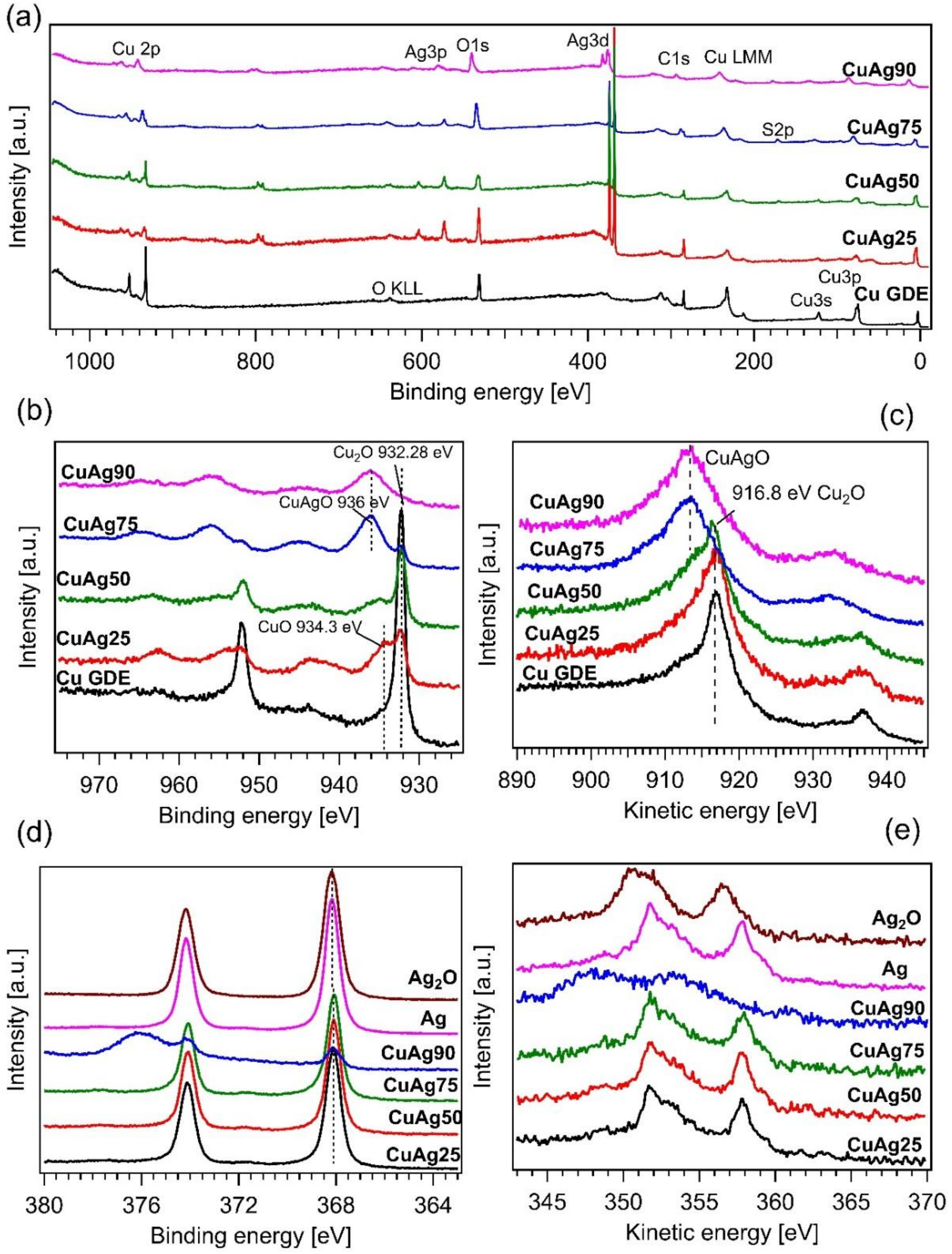

Figure S10. XPS spectra: (a) Survey, (b) Cu 2p, (c) Cu LMM Auger, (d) Ag 3d, and (e) Ag MNN Auger of Cu, CuAg-25, CuAg-50, CuAg-75, and CuAg-90 samples prepared on GDL. All spectra were acquired using a photon energy of 1144 eV.

The XPS survey spectra (Figure S10a) reveal that the samples comprise C, O, Cu, Ag and S elements, with sulphur originating from residual Ag_2SO_4 used during the synthesis. The Cu 2p XPS spectra (Figure S10b) indicate that pure Cu GDE exhibits a Cu $2p_{3/2}$ peak at a binding energy (BE) of 932.28 eV, consistent with the main presence of Cu_2O[1]. Introducing Ag atoms and increasing the preparation temperature up to 50°C, a new Cu $2p_{3/2}$ component emerges at 934.3 eV, accompanied by a distinct satellite feature, which indicates the formation of CuO, consistent with the literature[1]. At 75°C, a new peak appears at ~ 936 eV, which cannot be attributed to conventional CuO or Cu_2O phases. This spectral feature likely suggests the formation of a mixed Ag–Cu oxide phase, as a similar high BE component has been previously observed in mixed Cu oxide systems, such as $Ag_2Cu_2O_3$[2] or $CuAl_2O_4$[3]. This interpretation is further supported by the Cu LMM Auger spectra (Figure S10c), which exhibit broadening and energy shift at temperatures above 75 °C, indicating significant changes in the local chemical environment of Cu atoms.

In contrast, Ag 3d core levels (Figure S10d) and Ag MNN Auger spectra (Figure S10e) do not provide direct evidence for the formation of a mixed oxide phase. However, these results do not rule out the presence of a surface-enriched Ag layer interacting with the underlying Cu oxide matrix[4], or the formation of Ag–Cu bimetallic compounds[5]. In fact, the Ag 3d core level maintains the same linewidth of pure Ag or Ag_2O, but the corresponding Ag 3d5/2 peak in CuAg samples is centered at 368.1 eV, slightly lower than the 368.2 eV observed for pure Ag or Ag_2O. This result suggests the presence of electronic interactions and possible charge transfer between Ag and Cu species. However, at 90 °C both the Ag 3d and Ag MNN Auger spectra exhibit significant broadening and a pronounced shift in energy, indicating the formation of a less conductive phase. These spectral changes may arise from sample charging effects associated with the development of a new mixed or defective oxide phase with low electrical conductivity.

The apparent inconsistency between the Cu and Ag spectroscopic features is likely attributable to the differing probing depths at the same photon energy (1144 eV). The Ag 3d signal, due to the higher kinetic energy of its photoelectrons at the employed photon energy, is more bulk-sensitive than the Cu 2p and the corresponding XAS signals.

1.3 Electrochemical characterization

The electrocatalytic performance of the Cu-Ag electrodes towards CO_2RR was evaluated in a three-electrode three-compartment flow cell (Electrocell Europe A/S), as shown in Figure S11. The cathode was an as-prepared Cu-Ag electrode. The anode is a commercial Ir-coated Ti plate (Ir-MMO, ElectroCell Europe A/S). A mini-Ag/AgCl (3 M Cl^-, 1 mm, leak-free LF-1) was used as the reference electrode. A 1 M KOH (CAS: 1310-58-3, Sigma-Aldrich) aqueous solution was served as both anolyte and catholyte. 1 M KOH was chosen because it is conductive enough with respect to bicarbonate electrolytes and can maintain a high pH, which favours the C-C dimerization and suppresses HER. Further increase in KOH concentration could lead to significant loss of CO_2 through chemical reaction between CO_2 and OH^-. In this work, the gas flow reader in the outlet of CO_2 chamber indicates that 1 M KOH did not result in significant CO_2 loss since the gas flows at the inlet and outlet of the gas chamber were similar. An anion exchange membrane (Sustainion® 37-50, Dioxide materials) was used to separate the anolyte and catholyte chambers. At the cathodic side of the cell, the cathode separated the catholyte and CO_2 gas chambers, with both flows in a single-pass mode. At the anodic side, the anolyte passes through the chamber in a re-circulation mode. Liquid and gas flow rates were maintained at 3.5 mL/min and 25 mL/min, using a peristaltic pump and mass flow controller, respectively.

Galvanostatic tests were performed using a CH potentiostat (CHI760D) at current densities of 200, 400, and 600 mA cm^{-2}. The cathode potential was monitored over time thanks to the reference electrode. The reported potentials were rescaled and referred to the reversible hydrogen electrode (RHE) by using the Nernst equation (Eq. S2).

$$E\ (V_{RHE}) = E\ (V_{Ag/AgCl}) + E^0_{Ag/AgCl}\ (V_{SHE}) + 0.059 * pH\ (V) \qquad \text{(Eq. S2)}$$

where $E^0_{Ag/AgCl}$ is the standard potential of the reference electrode (0.22 V_{SHE}); E ($V_{Ag/AgCl}$) is the measured cathode potential against the reference electrode; pH is the pH value of the electrolyte; E (V_{RHE}) is the reported potential. Unless otherwise specified, all potentials are referenced to the RHE in this work.

HPLC (Nexera series, SHIMADZU) was used to detect and quantify the liquid products in the catholyte collected at the outlet of the catholyte chamber. Two liquid aliquots were collected at intervals of 120s and 3600s at the outlet of the cell to monitor the possible product changes during each test. An additional sample was also taken from the catholyte collection bottle at the end of each test to see the average production during the whole test. The samples were analysed using a column (rm9.h0.s3008, Repromer H, 9 µm, 300*8 mm, Dr. Maisch) with a 9 mM H_2SO_4 mobile phase and PDA and RID detectors.

Gas analysis was conducted in real time using a Micro Gas Chromatograph (Fusion®, INFICON), which is composed of two channels with a 10 m Rt-Molsieve 5A column and an 8 m Rt-Q-Bond column, respectively, and both with a micro thermal conductivity detector. Ar and He were used as carrier gases for the Molsieve 5A and Rt-Q-Bond columns, respectively, to separate H_2, CO, CH_4 and C_2H_4, C_2H_6, respectively.

Thanks to the extracted concentration of liquid and gas products, it is possible to calculate faradaic efficiency (FE) for all the products (Eq. S3). In this equation, the charge needed to produce a specific number of moles (N) of the product is divided by the total charge consumed during the corresponding reduction period (Q). Here, *n* represents the number of electrons required to form one molecule of the product (*n = 2* for CO, $HCOO^-$, and H_2; *n = 8* for CH_4 and CH_3COO^-; *n = 12* for C_2H_4 and C_2H_5OH), and *F* is the Faraday constant, equal to 96485 C mol^{-1}.

$$FE = \frac{nNF}{Q} \cdot 100\% \qquad \text{(Eq. S3)}$$

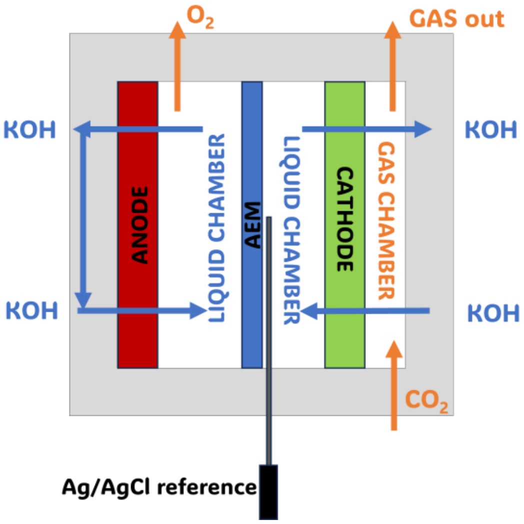

Figure S11. Schematic of the electrochemical flow cell.

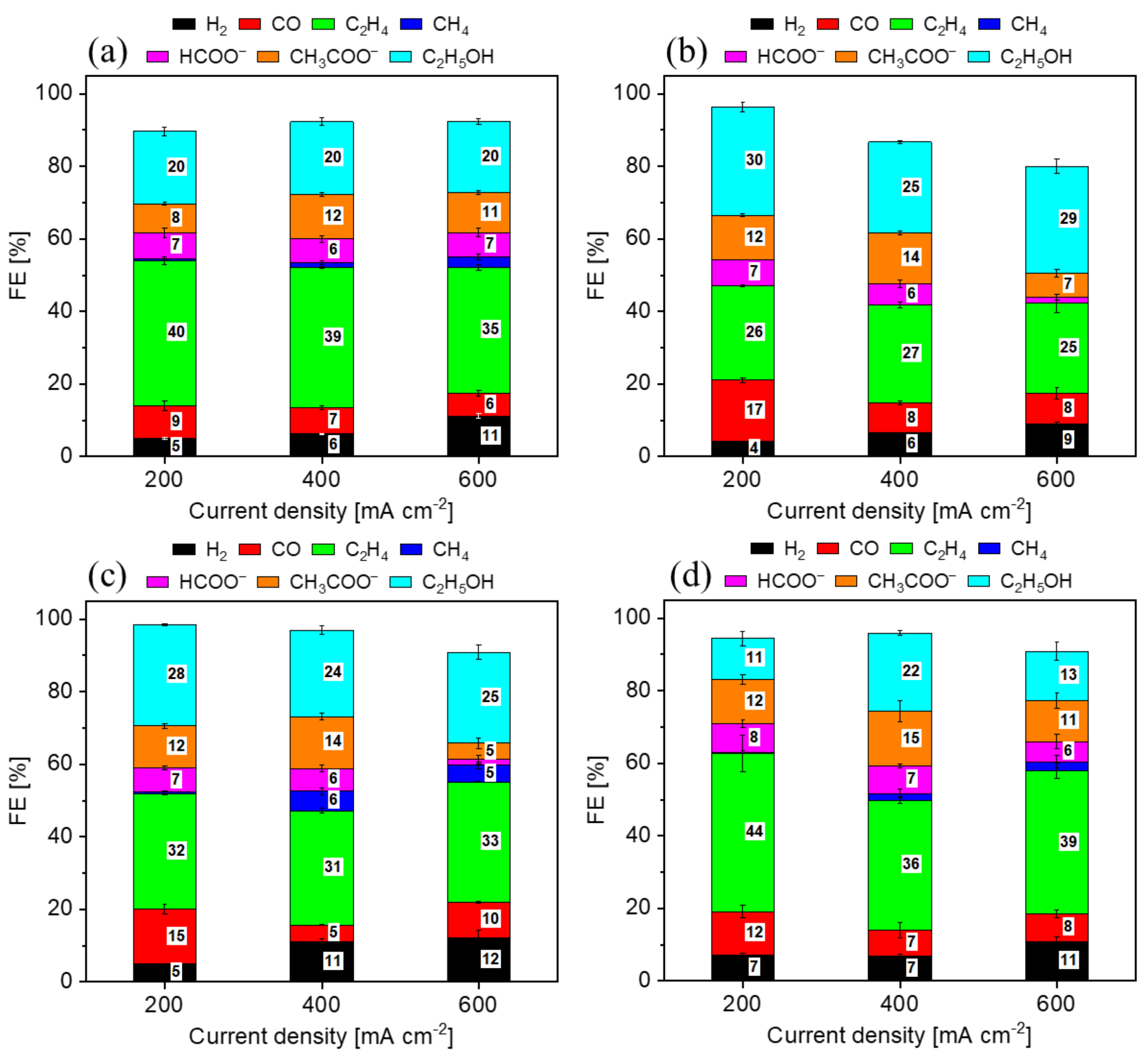

Figure S12. Resulting FE in flow cell set-up at 200/400/600 mA cm^{-2} for Cu-GDE (a), CuAg-25 (b), CuAg-50 (c) and CuAg-90 (d).

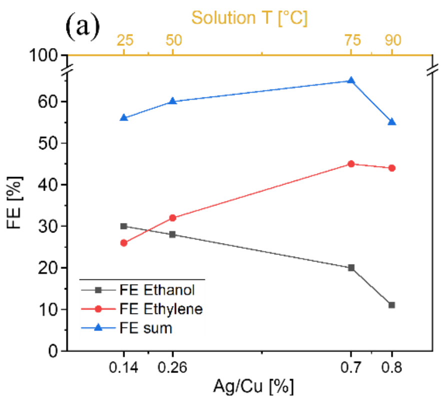

Figure S13. Faradaic efficiencies towards ethanol and ethylene at increasing Ag/Cu ratios (and solution temperatures) estimated at (a) 200mA cm^{-2}, (b) 400 mA cm^{-2} and (c) 600 mA cm^{-2}.

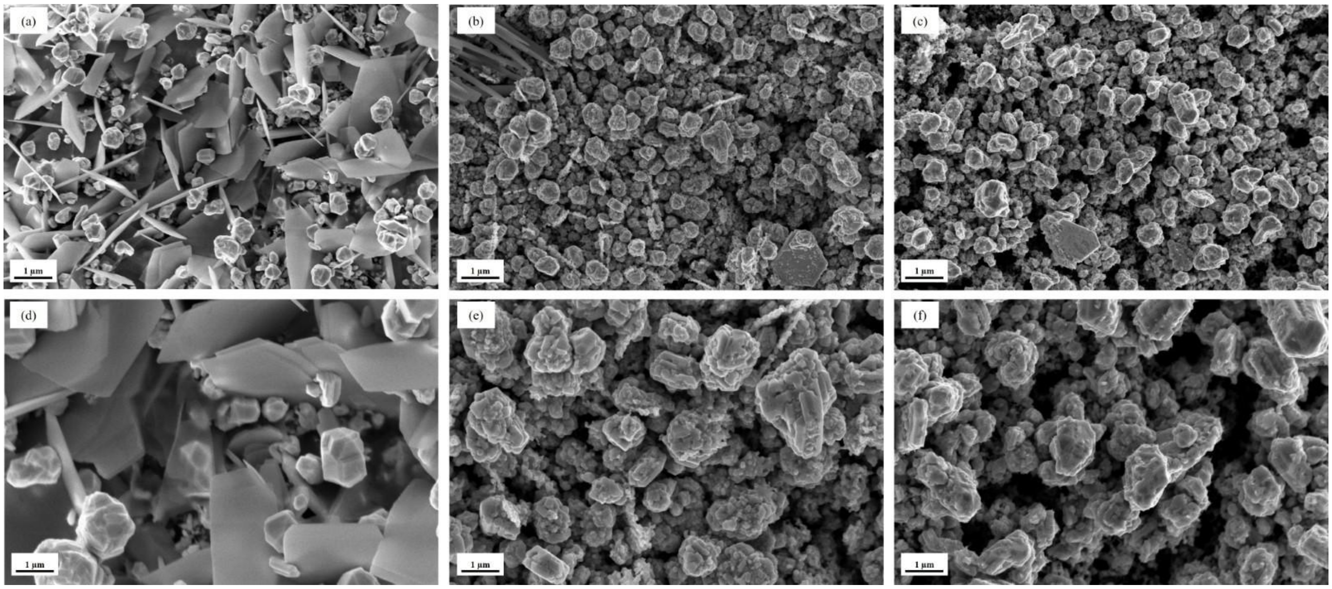

Figure S14. FESEM images of CuAg-75 at different moment of the 3-hour test: (a) fresh sample at 10K; (b) after 1 hour at 10K; (c) after 3 hours at 10K; (d) fresh sample at 25K; (e) after 1 hour at 25K and (f) after 3 hours at 25K.

1.4 In-situ XAS Characterization

The evolution of the electronic properties of selected Ag-Cu catalysts under negative potentials was studied by operando s-XAS using an advanced electrochemical setup developed at the BACH beamline. This technique provides insights into unoccupied electronic states, aiding in understanding the catalytic reaction mechanisms by monitoring the oxidation state variations of the catalyst and the transient species formation at solid/liquid interfaces.

This approach involves the confinement of the liquid electrolyte within an electrochemical cell (EC-cell, Fig. S15) equipped with a three-electrode configuration. A soft X-ray transparent membrane, consisting of a 100 nm thick, 1 mm^2 wide, Si_3N_4 membrane coated with 3 nm Ti and 15 nm Au layer on 1 cm^2 support (Au-Si_3N_4), serves both as a barrier between the vacuum environment and the liquid sample, and working electrode. The experimental setup includes a leak-less Ag/AgCl reference electrode, a platinum counter electrode positioned at the bottom cell compartment, and the Au-Si_3N_4 window as the working electrode, where the catalytic material is deposited as a thin film.

Two selected samples (Cu and CuAg-75 catalysts) were deposited onto the working electrode (Au-Si_3N_4 window) using the sputtering technique combined with galvanic displacement, as described above for Cu-Ag GDE. A spray coating of Nafion (1.5 wt%) was also applied to prevent delamination of the catalyst during operando-XAS experiment.

The operando s-XAS experiment was conducted by measuring the Cu L-edge and O K-edge in FY mode, using a photodiode (IRD, AXUV100G) placed at 25° with respect to the incident X-ray beam.

A 0.1 M $KHCO_3$ electrolyte solution, saturated with CO_2, was flowed through the cell using a peristaltic pump (DG10-BT103S). In-operando XAS spectra were recorded at different applied potentials, starting from the OCP to -1 V on both samples. Each potential was maintained for a duration of half an hour,

during which the Cu L-, O K-, and Cu L-edges were sequentially measured. This sequence was recorded to detect any change in the material oxidation state during the acquisition at well-defined voltages.

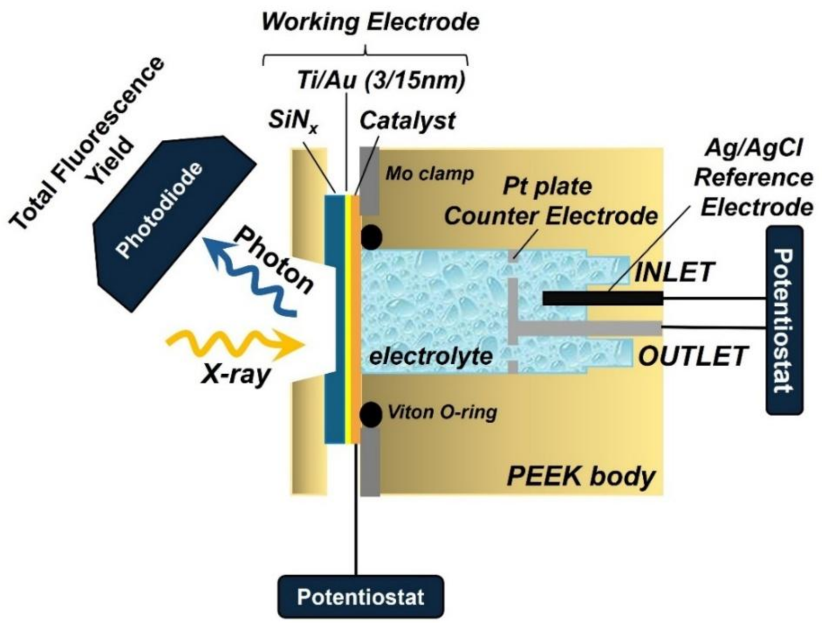

Figure S15. Scheme of the EC-cell and measurement setup for in situ s-XAS experiments implemented at the BACH beamline, Elettra Sincrotrone Trieste, reproduced from: Napal Azcona, I. X-Ray Spectroscopies for Hydrogen Based Economy (Doctoral Dissertation)[6]

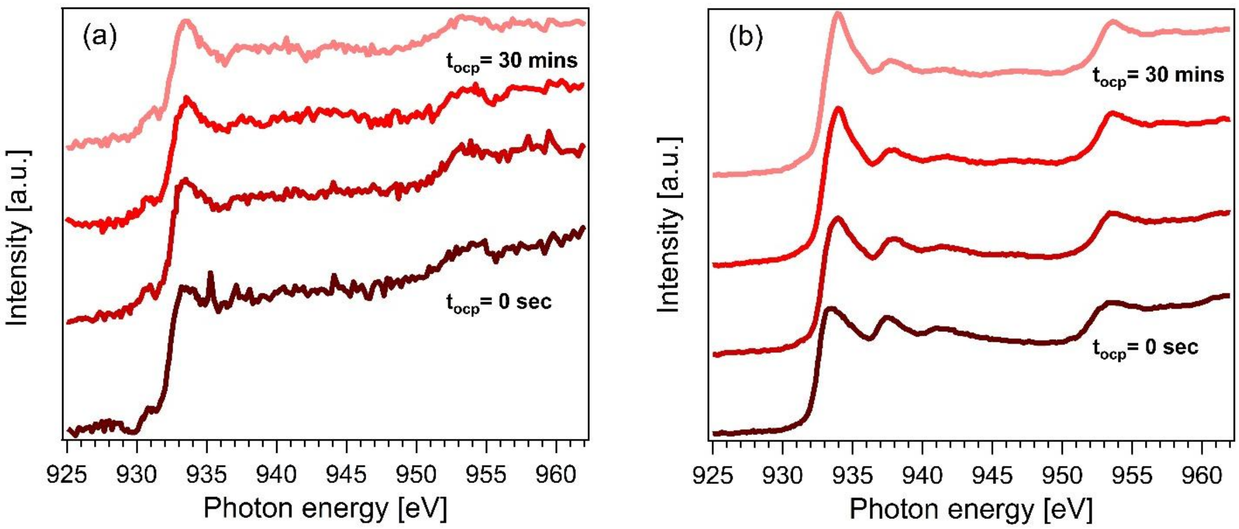

Figure S16. Cu L-edge spectra recorded in FY mode for (a) Cu and (b) CuAg-75 samples, showing their recovery to the OCP state after the operando XAS experiment.

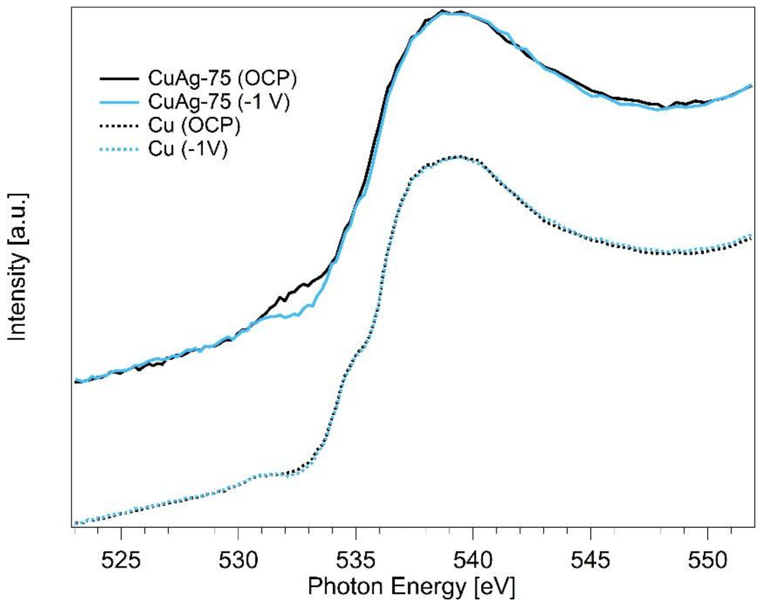

Figure S17. O K-edge spectra recorded in FY mode for Cu (dashed line) and CuAg-75 (continuous line) at the OCP (light blue) and at -1.0 V vs Ag/AgCl (black) during operando XAS experiment.

The O K-edge spectra exhibit a characteristic peak at 535 eV, which is typical of liquid water, thereby confirming the presence of the liquid electrolyte in the cell. The "bump" around 531 eV is an artifact caused by normalization with the reference current measured on the mirror before the experimental chamber. The O K-edge spectra of pure Cu do not show any evidence of a visible oxide, as its features may be partially hindered by window contamination. In contrast, the initial spectrum of the CuAg-75 sample at the open-circuit potential (OCP) reveals a shoulder around 532 eV, which can be attributed to the mixed AgCu oxide phase. This shoulder disappears at a potential of -1 V, indicating that the bimetallic catalyst is fully reduced.

Table S1. Ag/Cu ratios determined by EDX (over a wide rectangular area) on the CuAg samples prepared at different synthesis temperatures.

Sample	Cu [at.%]	Ag [at.%]	Ag/Cu
**CuAg-25**	14.6	2.0	0.14
**CuAg-50**	25.5	6.6	0.26
**CuAg-75**	16.9	11.8	0.70
**CuAg-90**	12.4	9.9	0.80

REFERENCES

(1) Torres-Ochoa, J. A., Cabrera-German, D., Cortazar-Martinez, O. et al. Peak-Fitting of Cu 2p Photoemission Spectra in Cu^0, Cu^{1+}, and Cu^{2+} Oxides: A Method for Discriminating Cu^0 from Cu^1. *Appl. Surf. Sci.* **2023**, *622*, 156960.

(2) Wang, H., Liu, Y. W., Li, X. Y. et al. Insights into Bimetallic $Ag_2Cu_2O_3$ Precatalyst for Electrochemical CO_2 Reduction to Ethanol. *ChemCatChem* **2024**, *16*(23), e202400992.

(3) Zhidkov, I. S., Belik, A. A., Kukharenko, A. I. et al. Cu-Site Disorder in $CuAl_2O_4$ as Studied by XPS Spectroscopy. *JETP Lett.* **2021**, *114*(9), 556–560.

(4) Herzog, A., Bergmann, A., Jeon, H. S. et al. Operando Investigation of Ag-decorated Cu_2O Nanocube Catalysts with Enhanced CO_2 Electroreduction toward Liquid Products. *Angew. Chem. Int. Ed.* **2021**, *60*(13), 7426–7435.

(5) Rollier, F. A., Muravev, V., Kosinov, N. et al. Cu–Ag Interactions in Bimetallic Cu–Ag Catalysts Enhance C_{2+} Product Formation during Electrochemical CO Reduction. *J. Mater. Chem. Mater. Energy Sustain.* **2025**, *13(*3), 2285–2300.

(6) Napal Azcona, I. X-Ray Spectroscopies for Hydrogen Based Economy (Doctoral Dissertation), Università degli Studi di Trieste, Italy, **2025**.
